# CuO Nanorods Immobilized Agar-Alginate Biopolymer: A Green Functional Material for Photocatalytic Degradation of Amaranth Dye

**DOI:** 10.3390/polym15030553

**Published:** 2023-01-21

**Authors:** Akshara Bassi, Kushal Qanungo, Imran Hasan, Alanoud Abdullah Alshayiqi, Alanood Sulaiman Ababtain, Fahad A. Alharthi

**Affiliations:** 1 Environmental Research Lab, Department of Chemistry, Chandigarh University, Mohali 140413, India; 2 Department of Chemistry, College of Science, King Saud University, Riyadh 11451, Saudi Arabia

**Keywords:** CuO nanorods, box-cox transformation, response surface methodology, biopolymers, photocatalytic degradation

## Abstract

The contamination of water is increasing day by day due to the increase of urbanization and population. Textile industries contribute to this by discarding their waste directly into water streams without proper treatment. A recent study explores the treatment potential of copper oxide nanorods (CuO NRs) synthesized on a green basis in the presence of a biopolymer matrix of agar (AA) and alginate (Alg), in terms of cost effectiveness and environmental impact. The synthesized bio nanocomposite (BNC) was characterized by using different instrumental techniques such as Fourier transform infrared spectroscopy (FTIR), X-ray diffraction (XRD), ultra-violet spectroscopy (UV-Vis), scanning electron microscopy-energy dispersive X-ray-elemental analysis (SEM-EDX), transmission electron microscopy (TEM), selected area diffraction pattern (SAED) and X-ray photoelectron spectroscopy (XPS). The optical studies revealed that immobilization of CuO NRs with Alg-Agar biopolymer blend resulted in an increase in light absorption capacity by decreasing the energy bandgap from 2.53 eV to 2.37 eV. The bio nanocomposite was utilized as a photocatalyst for the degradation of amaranth (AN) dye from an aquatic environment under visible light irradiation. A statistical tool known as central composite design (CCD) associated with response surface methodology (RSM) was taken into consideration to evaluate the optimized values of process variables and their synergistic effect on photocatalytic efficiency. The optimized values of process variables were found to be irradiation time (45 min), AN concentration (80 ppm), catalyst dose (20 mg), and pH (4), resulting in 95.69% of dye degradation at 95% confidence level with desirability level 1. The rate of AN degradation was best defined by pseudo-first-order reaction based on the correlation coefficient value (R2 = 0.99) suggesting the establishment of adsorption-desorption equilibrium initially at the catalyst surface then photogenerated ^•^O_2_^−^ radicals interacting with AN molecule to mineralize them into small non-toxic entities like CO_2_, H_2_O. The material used has high efficiency and stability in photocatalytic degradation experiments up to four cycles of reusability.

## 1. Introduction

With increasing population and development of industries, the contamination of water present at surface and ground level has become a global issue [1]. The organic dyes which are used in textile and food industries have become a center of research in environmental science because of their persistent, toxic and carcinogenic effects on human life [2,3]. Among all the dyes, the water soluble non-biodegradable azo dyes are ubiquitous; they contain one or more than one -N=N- group bonded to aromatic rings and different functional groups [4,5,6]. Amaranth dye is an anionic acidic monoazo dye with chemical formula C_20_H_11_N_2_O_10_S_3_, having the IUPAC name of trisodium 1-(4-supho-1-naphthylazo)-2-naphthol-3,6-disuphonic acid; it is widely used as colorant under the name of Acid Red 27 and Food Red 9 [7,8]. Contamination of water by the dye is extremely visible and can affect the water transparency, esthetics and gas solubility, and can block the penetration of light, affecting the aquatic life [9]. The dye has been proved to be toxic, carcinogenic, and mutagenic in the reproductive and immune system, so it has become a research priority to eliminate the dye from the environment. There are several techniques such as chemical oxidation, bioremediation, electrochemical degradation, adsorption and photocatalytic degradation reported in the literature to treat amaranth dye [10,11]. Among these methods, photocatalytic degradation associated with advanced oxidation processes has been showed to be a very promising technique for mineralization of the organic pollutants [12]. In photocatalysis, material as a catalyst absorbs the solar radiation which results in the generation of electron-hole pairs [13,14]. These photogenerated electron-hole pairs interact with surrounding water; the oxygen molecules will produce highly reactive superoxide and hydroxyl radicals responsible for the degradation process [15,16]. Due to its ease in operation, cost effectiveness and high efficiency of degradation, this method has gained applicability in different fields of engineering and technology such as hydrogen release via water splitting, environmental remediation as well as photo reducing CO_2_ and N_2_ [17,18,19]. Now there is a need to develop such types of catalyst materials which can absorb solar radiation efficiently and degrade organic pollutants.

Transition metal-based metal oxide semiconductor nanoparticles (NPs) have gained attention as photocatalysts due to their exceptional optical, electronic, catalytic, gas sensing, inexpensive, low band gap, non-toxic, thermal and chemically stable properties [20]. These materials generally have oxygen vacancies due to which they can change the energy bandgap [21,22]. There are different types of metal oxide NPs used in the photocatalysis techniques reported in the literature such as TiO_2_, ZnO, CuO, MgO, CeO_2_, V_2_O_5,_ etc. [23,24]. Among these, copper oxide (CuO), because of its abundance in nature, cost effective, non-toxic, environmentally friendly and biocompatible features has found a wide range of applications such as in sensors, supercapacitors, catalysis, field emission, antibacterial activity, optoelectronic devices, solar cells, pharmaceutics, photocatalysis, energy storage, adsorbent, antioxidant and anticancer activities [25,26,27,28]. They are generally classified as p-type semiconductors having an energy bandgap value of 1.2–1.4 eV. Copper oxide possesses two phases, i.e., cubic cuprous oxide (Cu_2_O) and monoclinic cupric oxide (CuO). In case of photocatalysis, CuO is preferred over Cu_2_O because of superior stability and a low band gap which allows optical band gap tuning by appropriate metals, applying to a wide range of visible radiation [29]. The reactivity of Cu as Cu^2+^ together with the low band gap value of CuO makes it a photocatalyst for the wastewater treatment [30]. The photocatalytic activities of CuO depend upon their charge separation properties but fast recombination of electron-hole pairs hinder their applicability to a wider range of applications [15]. To address this issue, surface functionalization by a biopolymer material was taken into consideration which further improved the optical properties of the nanomaterial due to synergistic effects [18]. The synergistic charge transfer from the functional groups of the biopolymer can efficiently improve the lifetime of photogenerated electrons through refining the properties of charge separation and hence improving the photocatalytic properties [31].

Biopolymers such as cellulose, chitosan, agar gum, alginate, starch, agarose and many more have been employed as sacrificial soft patterns to form the metal oxide nanoparticles [32,33]. The main advantages of biopolymer material with metal oxide as templates include mild reaction conditions, easy removal, easy scale up and simple synthesis [32]. Agar gum is a hydrophilic polysaccharide, a gelatinous substance which is present in red seaweed such as *Gelidium* and *Gracilaria*. It consists repeating units of galactose with β-1,3-linked-D-galactopyranose and α-1,4-linked-3,6-anhydro-L-galactopyranose [33]. Alginate is composed of 1,4-linked-α-L guluronic acid and β-D-mannuronic acid units in pyranose form, arranged in linear blocks [34]. It has high adsorption capacity, with accessibility of oxygen-containing functional groups, i.e., carboxyl and hydroxyl groups as attaching points [35].

Response surface methodology (RSM) has emerged as a statistical and mathematical technique for optimization of the effect of process variables and setting up a design for experiments [36]. It reduces the number of trials in the experimental design and highlights the synergistic effect of two or more variables on the outputs of the reaction simultaneously [37]. In the present study we have applied the central composite design (CCD) associated RSM model to optimize the irradiation time (A), pH (B) and AN concentration (C) for the photocatalytic degradation of AN dye by CuO/Alg-Agar BNC.

## 2. Materials and Methods

### 2.1. Chemicals Used

Low molecular weight sodium alginate SLR was purchased from Sigma-Aldrich, Mumbai India. Agar gum was purchased from Fluka, Chennai, India. Copper nitrate [(Cu(NO_3_)_2_·5H_2_O) hemi pentahydrate 98%] was purchased from Merck, Banglore, India. Amaranth was purchased from Otto Chemie Pvt. Ltd., Mumbai, India. These chemical materials were used without any purification, modification or decontamination. A 1000 mg L^−1^ standard solution of amaranth was prepared by dissolving an appropriate amount of dye in 100 mL of double distilled water.

### 2.2. Synthesis of CuO/Alg-Agar BNC

The proposed BNC material was synthesized by a simple one pot chemical coprecipitation reaction with some modification [20]. In a three-necked round bottom flask of 100 mL, a ratio of 1:1 (volume%) alginate and agar gum were dissolved until appearing homogeneous. This solution was added drop wise to a 50 mL of 1.2 M solution of Cu(NO_3_)_2_·5H_2_O and the reaction was left on magnetic stirring followed by addition of 25% ammonia solution to maintain the pH of the reaction media as 8–9. After 12 h, the product was filtered and washed several times with deionized water and dried in a hot air oven at 60 °C for 7 h.

### 2.3. Characterization of CuO/Alg-Agar BNC

The type of crystalline structure, bonding and different surface morphology of synthesized bio nanocomposites was characterized by using various analytical instruments within the average size of nanomaterial in a biopolymer matrix. The functional group and type of bonding between CuO and Alg–Agar was determined by Fourier transform infrared (FTIR) spectroscopy using a PerkinElmer PE1600 spectrophotometer (Massachusetts, USA) in the range of 400–4000 cm^−1^. A Rigaku Ultima 1 V X-ray diffraction (XRD, Austin, TX, USA) diffractometer was utilized to collect the information about the crystalline structure of CuO NPs with respect to AA/ALG moieties. Scanning electron microscopic (SEM) was used for the determination of surface morphology, elemental identity, chemical composition and homogeneity of BNC, combined with electron dispersive X-ray analysis (SEM-EDX) (SEM; JEOL GSM 6510LV, Tokyo, Japan). The particle size and dispersal of nanocomposite in the biopolymer blended matrix was observed with a transmission electron microscope (TEM: JEM 2100, Tokyo, Japan). Selected area electron diffraction (SAED) designs were performed by using aperture that selected a 200 nm diameter area of TEM section; d-spacings of diffraction patterns were calibrated using d-spacings of gold determined under identical conditions.

### 2.4. Response Surface Methodology (RSM) and Experimental Design

The experimental design, mathematical modeling and optimisation were constructed using Desgin Expert that was later implemented via RSM-coupled CCD to check the synergistic or antagonistic effects on any two or more than two variables and the response of the nanoparticles [36]. The complete design consisted of three factors including (i) irradiation time A, (ii) ph B and (iii) concentration each at five levels (−2, −1, 0, +1, +2) as shown in Table 1. For the prediction of degradation of AN on the CuO/Alg-Agar BNC, the three variables can be expressed by using a quadratic regression model as given below [37].

(1) $$y=b_{0}+\sum_{i=1}^{n}b_{i}x_{i}+\sum_{i=1}^{n}b_{ii}x_{i}{}^{2}+\sum_{1\leq i<j}^{n}b_{ij}x_{i}x_{j}+\varepsilon$$

where *y* is response of design, *b_o_* is model constant, *b_i_* is quadratic constant, *b_ij_* is interaction constant, *n* is number of variables used in design, ***ε*** is residual statistical term, *x_i_* and *x_j_* are linear functions that convert original actual values, X_i_ − αC_i_, X_i_ − C_i_, X_i_, X_i_ + C_i_, and X_i_ + αC_i_ to coded values −α, −1, 0, +1 and α [38].

(2) $$x=\frac{\left(X-x_{i}\right)}{C_{i}}$$

### 2.5. Photocatalytic Activity

To perform the photocatalytic experiment, 20 mL of aliquot with a specific concentration of AN was placed in a 50 mL conical flask and agitated with a sonicator, in the presence of visible radiation, disbursing the given quantity of irradiation time/ph/concentration which was recommeneded by the RSM-CCD design. Then the final AN concentrations after completion of the experiments were analyzed using UV–Vis spectrophotometer and photodegradation capacity. Percentage degradation at maximum absorption wavelength was given as below-

(3) $$\mathrm{Degradation}\;(\%)=\frac{C_{o}-C_{t}}{C_{o}}\times 100$$

where C_o_ and C_t_ are the initial and final concentrations of AN after time t, respectively.

## 3. Results and Discussion

### 3.1. Characterization of CuO/Alg-Agar BNC

**Figure 1:**
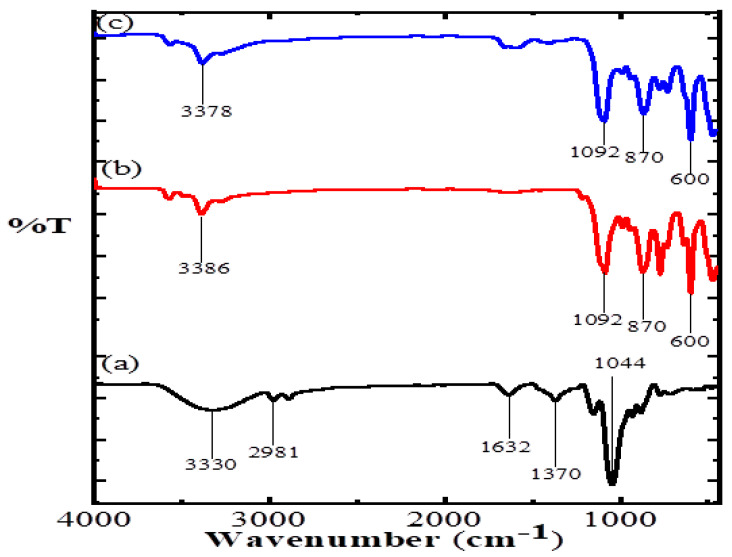
FTIR spectrum of (**a**) Agar agar, (**b**) CuO and (**c**) CuO/Alg-Agar BNC.

The FTIR spectra and their vibrational frequency data of AA, CuO and CuO/Alg-Agar BNC are shown in Figure 1. Table 2 shows the frequency of specific functional groups present. The FTIR analysis has vibrational frequencies which suggest the monoclinic phase, and stabilization of Cu^2+^ into CuO by molecular interface of groups to create a Alg–Agar–O–Cu type lattice.

**Figure 2:**
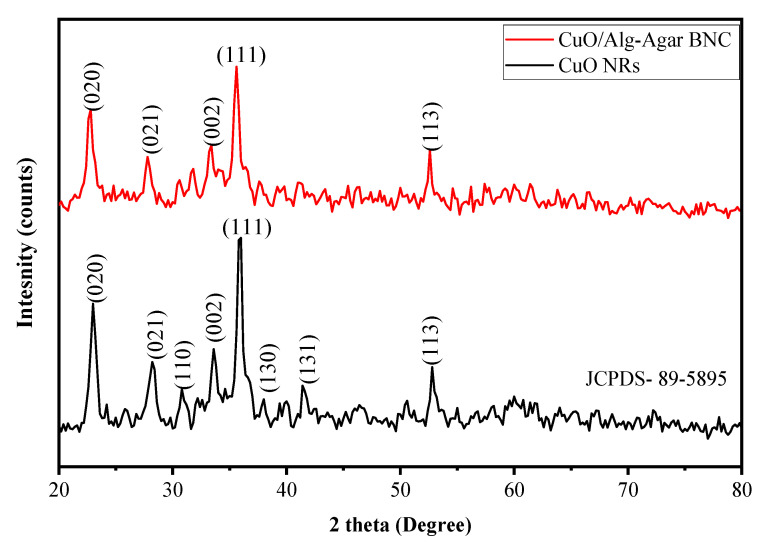
XRD spectra of CuO NRs (black line) and CuO/Alg-Agar BNC (red line).

The data about the lattice and solid structure of the synthesized material CuO/Alg-Agar BNC were obtained by XRD analysis, which gave spectra as shown in Figure 2 (red line). Peaks at 22.7°, 27.8°, 33.3°, 35.6° and 52.6° belong to Miller indices values of (020), (021), (002), (111) and (113). Figure 2 (black line) shows XRD spectra of CuO NRs; distinctive peaks at 2ϴ values of 22.7, 27.8, 30.7, 33.3, 35.6, 37.9, 41.3 and 52.6 belong to Miller indices values of (020), (021), (110), (002), (111), (130), (131) and (113) (JCPD-89-5895) [41]. Thus, comparison of the Miller indices values from CuO NRs with those for CuO/Alg-Agar BNC confirms that CuO NRs are effectively functionalized on the biopolymer matrix of Alg–Agar. Using Equation (4), the bulk CuO NRs are found to 56% crystalline and 44% amorphous whereas CuO/Alg-Agar BNCs were 21% crystalline and 79% amorphous in nature. The BNCs are amorphous in nature because the biopolymer matrix of Alg–Agar is capped around the CuO NRs.

The information about crystalline size and %crystallinity can be calculated using Equations (4) and (5) [42].

(4) $$D=\frac{0.9\times \lambda }{\beta \times \cos\theta }$$

(5) $$\%\mathrm{Crystallinity}=\frac{\mathrm{Area}\;\mathrm{under}\;\mathrm{the}\;\mathrm{crystalline}\;\mathrm{peak}}{\mathrm{Total}\;\mathrm{area}}?\;00$$

with *β* as half width of highest intensity peaks, λ as wavelength used (1.54 Å), *θ* as the angle of diffraction and *D* as crystalline size. Using Scherrer equation and XRD analysis, the crystal size of CuO NRs is found to be 12.11 nm whereas the crystal size of synthesized CuO/Alg-Agar BNC compound was found to be 10.82 nm.

**Figure 3:**
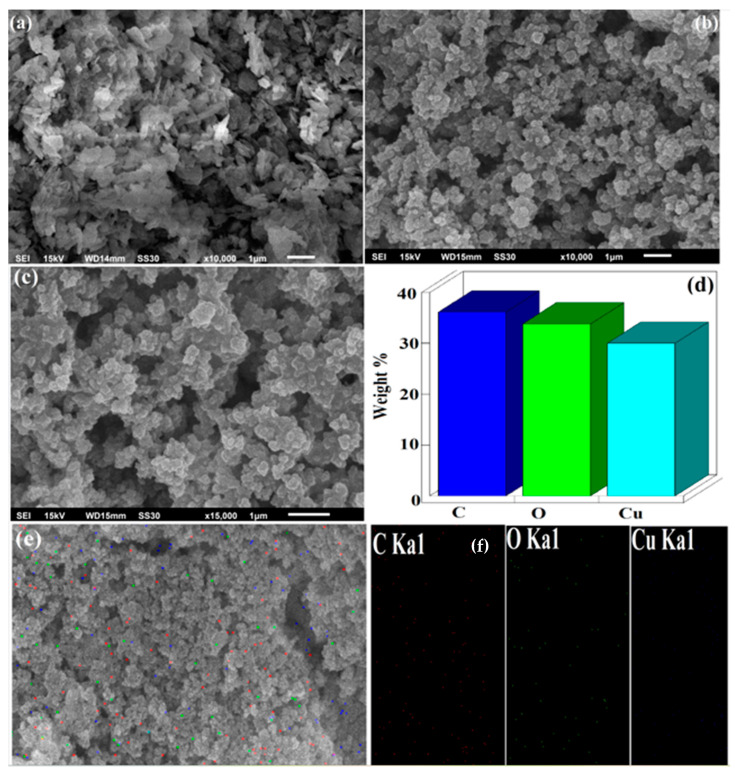
SEM image of (**a**) CuO NRs, (**b**,**c**) CuO/Alg-Agar BNC at 10,000 and 15,000 magnification range, (**d**) EDX spectra of CuO/Alg-Agar BNC, (**e**) selected area of SEM image of CuO/Alg-Agar BNC with (**f**) elemental mapping.

Surface morphologies are used to find the distribution of particles and their location in the biopolymer matrix during solid-state reactions. Figure 3a represents the SEM image of CuO NPs which suggest the tiny rods or needle like morphology. Figure 3b,c shows the SEM images of CuO/Alg-Agar BNC at 10,000 and 15,000 magnification which indicates an irregular shaped immobilized or coated morphology. Figure 3d represents the EDX spectra for CuO/Alg-Agar BNC which con-firms the presence of C, O, Cu in the material with composition of 59.01, 34.77 and 6.22 atomic percent. Figure 3e,f show elemental mapping results of C, O and Cu, which support the EDX results and show a significant distribution of CuO NRs on the biopolymer matrix.

**Figure 4:**
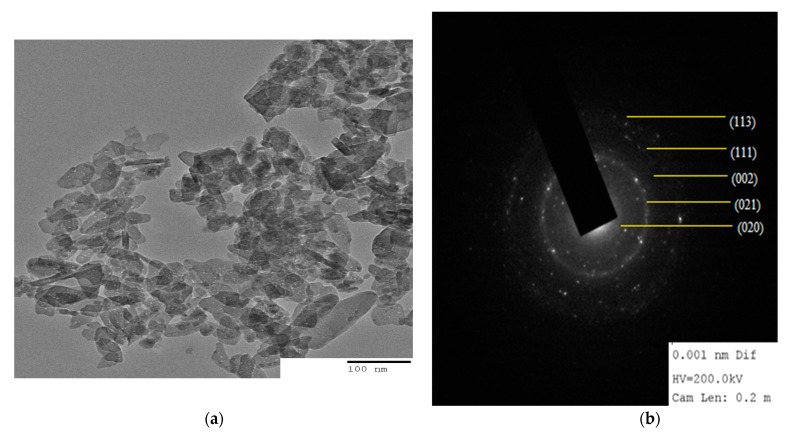
(**a**) TEM image of CuO/Alg-Agar BNC showing distribution of CuO NRs on the biopolymer matrix of AA-Alg and (**b**) SAED image of CuO/Alg-Agar BNC.

The size of particle and distribution in the matrix given by TEM are shown in Figure 4a,b. TEM image (Figure 4a) shows the finely distributed tiny rod or needle shaped CuO NRs in the locality of the biopolymer matrix with average diameter size of particle is 15.78 ± 0.95 nm which is close to the crystal size calculated by using XRD analysis. In the image, there were some aggregations, due to the presence of Alg-Agar biopolymer matrix which is responsible for the stabilization and reduction of CuO NPs. In Figure 4b, material was characterised by selected area diffraction pattern (SAED); it was found that each element is well distributed over the whole sample. On SAED, the diffraction peaks match the XRD pattern which proves the sample is amorphous in nature.

**Figure 5:**
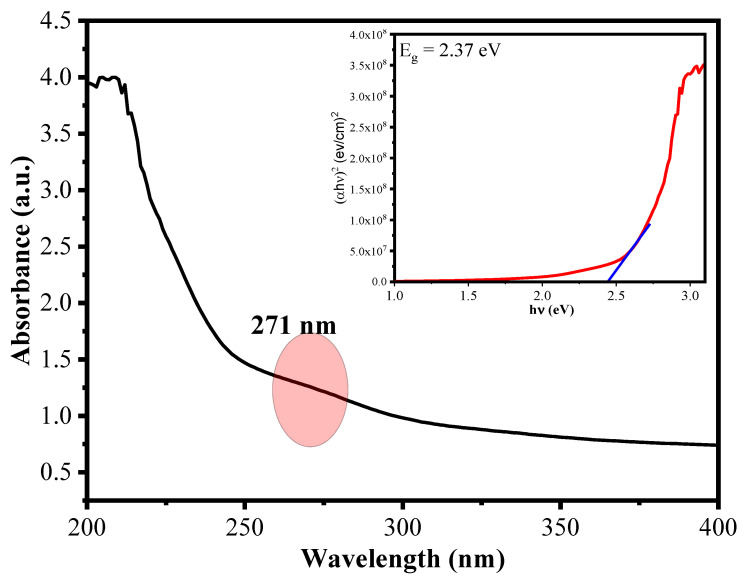
UV–Vis plot of CuO/Alg-Agar BNC having wavelength range (200–400 nm) and inset showing Tauc’s plot for calculating the energy band gap value for the material.

The optical absorption, energy band gap and embedding of Cu^2+^ ions into CuO NRs in the matrix of polymers of AA-Alg were evaluated by using UV-Vis spectroscopy. Samples were observed at wavelength range of 200–800 nm using a Shimadzu UV-1900 spectrophotometer. It can be seen from Figure 5 that The synthesized material CuO/Alg-Agar BNC shows an absorption peak (λ_max_) at 271 nm. From the literature, the absorption peak of CuO is found at 273 nm [43]. Thus, a blue shift was observed in CuO which suggests that CuO NPs are functionalized with the biopolymer matrix. Further, the energy band gap value is calculated by using Tauc’s plot equation given below [44]:

(6) $$(\alpha h\nu )=A(h\nu -E_{g}{)}^{n}$$

where α is the absorption coefficient, *h* is Plank’s constant, *v* is frequency of different radiations, A is constant and n is constant (value depends on type of band transitions: ½ is direct transitions and 1 is indirect transitions). CuO has direct transition and the value of n is 1/2 [45]. The inserted graph in Figure 5 shows the graph relating (αhv)^2^ and hv. The band gap from Tauc’s pot is 2.37 eV of synthesized CuO/Alg-Agar BNC. From the literature, E_g_ of CuO was found at 2.53 eV; the decrease in band gap because of contraction in quantum confinement results in the stability of the CuO with Alg-Agar biopolymer matrix.

**Figure 6:**
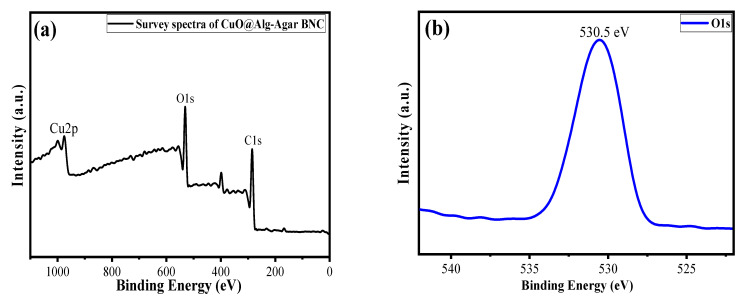

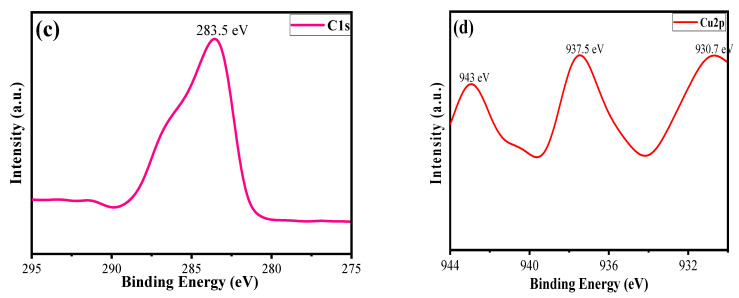
XPS spectra of (**a**) survey scan of CuO/Alg-Agar BNC, (**b**) O1s, (**c**) C1s and (**d**) Cu2p.

The oxidation state and chemical composition of each element in the synthesized material were evaluated by using X-ray photoelectron spectroscopy (XPS) analysis [46]. Figure 6a shows a survey scan of the CuO/Alg-Agar BNC, suggesting that Cu, C and O are main elements of the synthesized material. Figure 6b shows the O1s spectrum at binding energy 530.5 eV which corresponds to the surface oxidation. Figure 6c shows the C1s spectrum at binding energy 283.5 eV which corresponds to the C-C functional peak [47]. Figure 6d shows the Cu spectrum with binding energy peaks at 943 eV, 937.5 eV and 930.7 eV. These correspond to Cu 2p3/2, confirming the existence of CuO with +2 oxidation state [48].

### 3.2. RSM and Statistical Analysis Approach

The design contains 20 experimental patterns based on four variables such as irradiation time (A), pH (B) and AN concentration (C) via Design Expert. These variables have ranges such as irradiation time (25–50 min), pH (2–6) and AN concentration (26–94 ppm) as given in Table S1. On the basis of experimental and theoretical results, the quadratic regression model was engaged to determined the response of the coded values for three different variables regarding their interaction during the process. The quadratic equation may be expressed as given below [49]

R1 = +91.30 + 0.8885 × Irradiation time − 0.8298 × pH + 3.40 × AN Conc − 0.1937 × Irradiation time × pH − 0.4520 × Irradiation time × AN Conc − 0.2066 × pH × AN Conc − 0.3254 × (Irradiation time)^2^ + 1.27 × (pH) [2] − 1.63 × (AN Conc)^2^ (7)

In the above equation, positive signs indicate synergistic effects and negative signs indicate antagonistic effects. From the equation, irradiation time and concentration are pragmatic [50]. This suggests that absorption of AN by CuO/Alg-Agar BNC can be enhanced by expanding these components.

#### 3.2.1. Analysis of Variance (ANOVA)

The statistical implications and interaction results of individual terms found from the quadratic model for AN photodegradation were analysed by ANOVA. This implications of the regression model and each coefficient term were evaluated by using F and *p* values using Fisher’s null hypothesis which says that higher values of F and lower values of *p* confirm that the model’s appropriatness via RSM-coupled CCD. The appropriate conditions based on Fisher *p* > F < 0.05 are given in Table 3 [51]. The finding of *p* > F value of 0.0035 indicates that the proposed quadratic regession models have statistical significance and are applicable for the photocatalytic degradation of AN on the CuO/Alg-Agar BNC.

%degradation = +91.30 + 0.8885 × Irradiation time − 0.8298 × pH + 3.40 × AN Conc − 0.1937 × Irradiationtime × pH − 0.4520 × Irradiation time × AN Conc −0.2066 × pH × AN Conc.(8)

#### 3.2.2. Three Dimensional Response Surface Morphology and Its Interpretation

**Figure 7:**
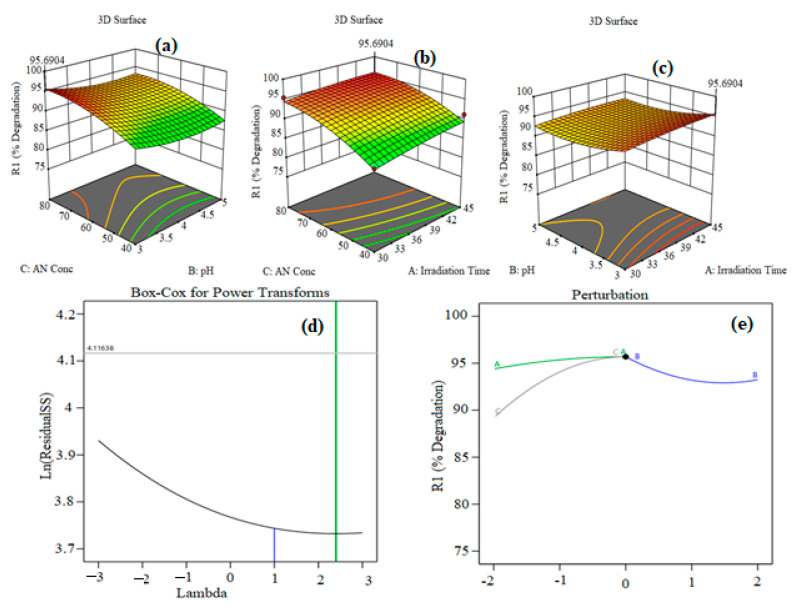
3D surface interactive plots of (**a**) AN concentration vs. pH, (**b**) AN concentration vs. irradiation time, (**c**) pH vs. irradation time, (**d**) Box–Cox for power transformation and (**e**) pertubation curve.

Figure 7a–e show the 3D response surface plot of the quadratic regression equation which explains the synchronous effect of any two variables on photocatalytic degradation when other variables are constant. Figure 7a shows the 3D graph of effect of pH and irradiation time on the photodegradation capacity of AN with different catalyst doses and different AN concentrations. From Figure 7a, it can be seen that with increases in pH, the degradation of AN also increases up to a certain point, while it then declines somewhat with furter increases in pH. A long irradiation time and high value of pH support the degradation of AN by CuO@AAALG nanoparticles (Figure 7a). There are large active pores present on the surface which facilitate the widespread interaction between the host and guest. During the reaction, active sites are increasing and become engaged in the degradation of AN, which results in the higher percent of degradation under the longer irradiation time. It was observed from the data that an increase in irradiation time from 30–45 min with pH from 3–5 results in a 79-95% increase of photodegradation capacity. This is due to the large number of hydroxyl radicals formed at high pH and the presence of high negative charge density on the catalyst surface, which attracts the AN azo dye via electrostatic interactions. Similarly, as shown in Figure 7b, there is an increased percent effectiveness of AN degradation as the concentration of AN increases from 60–80 mg L^–1^ as time increases from 30–45 min. With a further increase in the concentration of AN, the degradation percentage decreases as there is saturation of external sites on catalyst surface by dye molecules. Therefore, an optimized AN concentration was found to be 80 mg L^–1^ for the kinetics and thermodynamics studies. Figure 7c shows that as AN concentration increases, degradation capacity also increases. This enhanced photocatalytic efficiency enables the combination of more guest molecules by chemical interaction with active sites on the surface under radiation. This illustrates that a higher AN concentration favors improved percentage degradation.

Figure 7d represents Box–Cox power transformation, used to recover the normality of residuals from a quadratic model. Basically, Box–Cox transformation helps to normalize the data which is not properly distributed, using a suitable exponent, i.e., λ. Λ represents power where all the data should be elevated. The Box–Cox method was initially proposed as a solution for modifying homogeneity, linerarity, and normality [52]. Figure 7d shows the Box–Cox for power transformation plot of residuals. The blue line represnts the λ value for residuals as 1, whereas the suggested value for λ (green line) is 2.5. Thus, Box–Cox for power transformation is required for normal distribution of residuals. Figure 7e shows the perturbation curve for the interface between the three variables of irradiation time, pH and AN concentration. The perturbation plot is used to examine the most significant factor in the response. Curvature in the plot shows that the response is sensitive to particular variables, whereas flat lines show the response is insensitive to change in those particular variables [53]. The flat line for irradiation time shows that AN degradation efficiency was insensitive to this factor. On the other hand, the curvature of pH and concentration reveals that AN degradation efficiency was sensitive to this factor.

### 3.3. Kinetic Studies of Photodegradation

**Figure 8:**
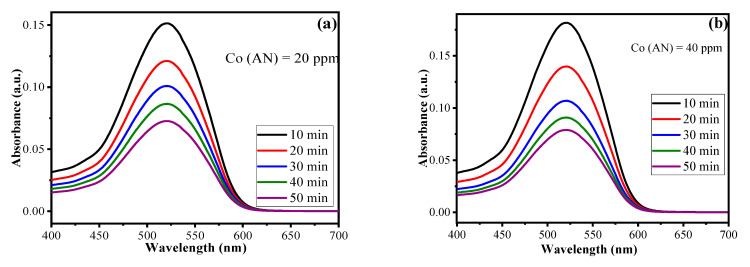

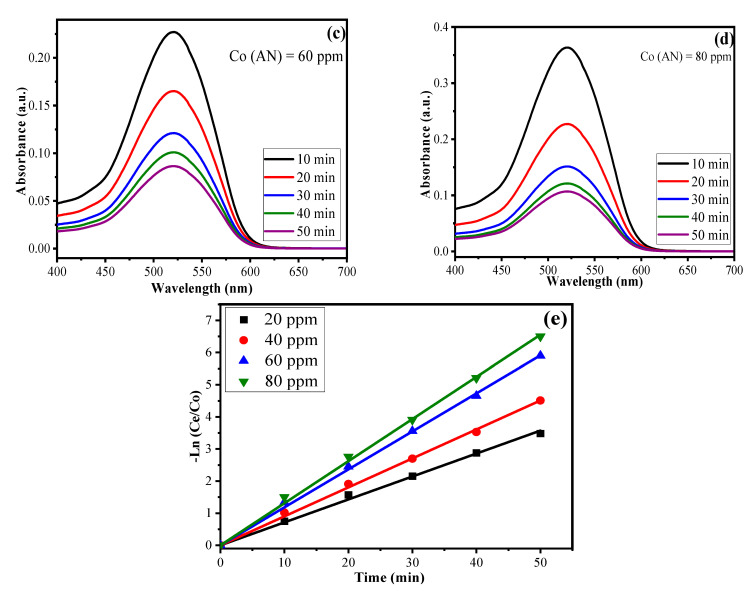
UV-Vis absorption spectra of AN at (**a**) 20 ppm, (**b**) 40 ppm, (**c**) 60 ppm, (**d**) 80 ppm under range of irradiation time 10–50 min at pH at 4 and (**e**) linear regression plot for pseudo first order kinetics.

The amount of AN degraded by using CuO/Alg-Agar BNC was investigated by using UV-Vis spectroscopy. To calculate the kinetics, data from irradiation time was utilised; a time-dependent experiment was performed by using different parameters coupled by the RSM-CCD model, namely, catalyst dose (0.02 g), pH (7.0), and irradiation time (10–50 min), using a 20 mL of aliquot of different AN concentrations (20, 40, 60, 80 mg L^–1^ of AN) and maximum absorbance at 520 nm. The photodegradation of AN was experimentally performed by using UV-Vis spectroscopy. As shown in Figure 8a–d, with the decrease in absorbance value, and as the irradiation time increases from 10–50 min, the color of the aliquot changes from red to colorless. The reason is that when light radiation interacts with the catalyst surface, transitions of electrons from VB to CB take place, resulting in the generation of reactive oxidant species such as ^•^OH/^•^O_2_^−^ radicals which degrade the AN dye molecules. Table S2 gives the data of absorbance vs. irradiation time at different AN concentrations. As the AN concentration is increased, the absorbance also increases. With increase of irradiation time, the photocatalytic efficiency of synthesized material decreases. The is a decrease of photocatalytic activity of AN at higher concentrations because of the screening effect formed by layers of AN molecules which delay the access of visible radiation to the catalyst surface, slowing the rate of ROS production. The mathematical equations for calculating the rate constant values and half-life period for pseudo first order kinetics are given below [54]

(9) $$-\ln(\frac{C_{t}}{C_{o}})=k_{1}t$$

(10) $$t_{1/2}=\frac{0.693}{k_{1}}$$

Here, *C_o_* and *C_t_* represent initial and final AN concentration respectively, *k*_1_ is the pseudo first order reaction constant, *t* is time and t_1/2_ represents the half-life period of reaction (min). Table 4 summarizes the values for *k*_1_, *R*^2^ and *t*_1/2_. The plot of *t* vs. −ln(*C_t_/C_o_*) gives a straight line (Figure 8e) and proves pseudo first order kinetics. The values acquired for *k*_1_ are 0.06, 0.09, 0.11, 0.13 min^−1^ corresponding to AN concentration for 20 ppm, 40 ppm, 60 ppm and 80 ppm, respectively, for CuO/Alg-Agar BNC. The kinetics suggest the photocatalysis of AN shows high efficiency at higher concentrations. The reason behind the masking effect is the formation of radicals as higher numbers of dye molecules are present at the solid-liquid interface.

### 3.4. Scavenger Effect and Degradation Mechanism

Reactive oxidant species (ROS) are mainly responsible for photodegradation of the organic pollutants into non-toxic units. The scavenger experiments were achieved by taking 25 mL aliquot part of 80 mg L^−1^ at 4 pH with 20 mg of CuO/Alg-Agar BNC for 45 min in presence of visible radiation. The separate aliquot samples were mixed with 3 mM of diffferent scavengers such as oxalic acid for h^+^_VB_, ascorbic acid for ^•^O_2_^−^ radicals, methanol for OH. radical and sodium sulphate for electron (e_CB_^−^) [55,56,57,58].

**Figure 9:**
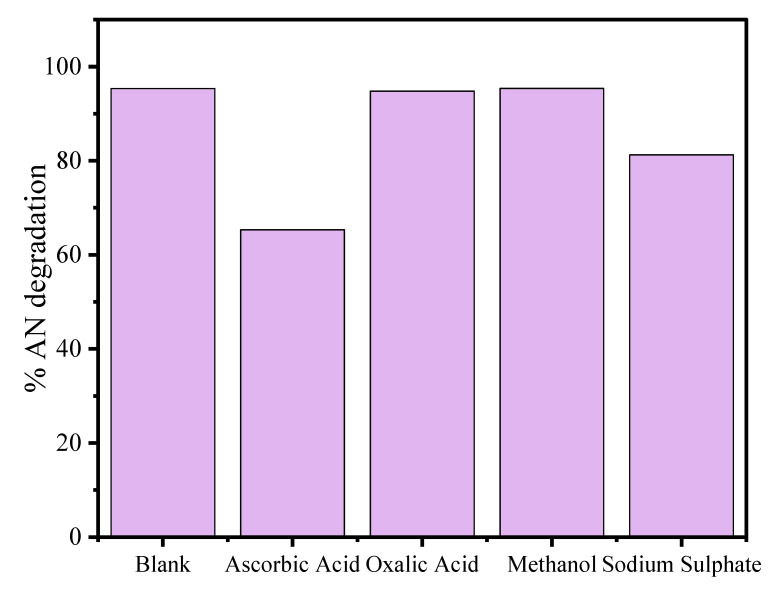
Scavenger test on the AN degradation by CuO/Alg-Agar BNC for determination of ROS.

The obtained results are given in Figure 9 and suggest that the photocatalytic efficiency of the material was mostly suppressed in the presence of ascorbic acid followed by sodium sulphate, indicating the involvement of ^•^O_2_^−^ and e^−^ in the mineralization of AN. A plausible mechanism of photodegradation of AN by CuO/Alg-Agar BNC material is given below.

(11) $$\mathrm{CuO}@\mathrm{Alg}-\mathrm{Agar}+h\nu \to \mathrm{CuO}@\mathrm{Alg}-\mathrm{Agar}(h_{\mathrm{VB}}^{+}+e_{\mathrm{CB}}^{-})$$

(12) $$O_{2}+e_{\mathrm{CB}}^{-}\to {}^{•}O_{2}^{-}$$

(13) $${}^{•}O_{2}^{-}+H^{+}\to {\mathrm{HO}}_{2}^{•}$$

(14) $$\mathrm{CuO}@\mathrm{Alg}-\mathrm{Agar}(e_{\mathrm{CB}}^{-})+{\mathrm{HO}}_{2}^{•}+H^{+}\to H_{2}O_{2}$$

(15) $$\mathrm{CuO}@\mathrm{Alg}-\mathrm{Agar}(e_{\mathrm{CB}}^{-})+H_{2}O_{2}\to \;{}^{•}\mathrm{OH}+{\mathrm{OH}}^{-}$$

(16) $$\mathrm{AN}+{}^{•}O_{2}^{-}\to \mathrm{AN}-\mathrm{OO}\;\to H_{2}{O+\mathrm{CO}}_{2}+\mathrm{etc}.$$

When visible radiation falls on the CuO/Alg-Agar BNC surface, the electrons-holes are formed (e_CB_^−^ + h_VB_^+^) in their conduction band and valence band, respectively. Then, the appeareance of O_2_ will trap the e_CB_^−^ electrons and form ^•^O_2_^−^ (superoxide radical), which reacts with H^+^ ions resulting in the formation of HO_2_^•^ radical. The holes h_VB_^+^ present on the surface of the catalyst will combine with H_2_O generating ^•^OH hydroxyl radicals. These and other reactive oxidant species are responsible for the degradation of AN which results in the generation of small non-toxic products such as H_2_O, CO_2_ and other products.

### 3.5. Reusability Test

**Figure 10:**
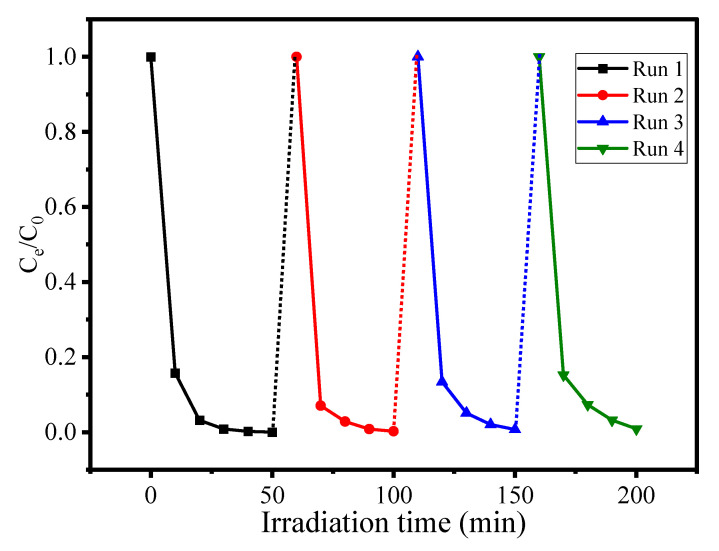
Reusability test for CuO/Alg-Agar BNC towards AN dye using 25 mL aliquot part of 80 mg L^–1^ at 4 pH with 20 mg of CuO/Alg–Agar BNC for 45 min in presence of visible radiation.

The stability and reusability test is an important parameter to evaluate the efficiency of the catalytic material in repeated cycles. Experiments were carried out using optimized conditions for AN degradation using CuO/Alg-Agar BNC under visible light radiation. After completion of the reaction, the material was separated by centrifuge and the supernatant was tested by UV-Vis spectrophotometer. The calculated photocatalytic efficiency after the first cycle was found to be 95.32%. The collected material was again washed with distilled water and ethanol and dried in an oven at 40 °C for 4 h. The material was again tested against AN dye under photocatalytic conditions and the photocatalytic efficiency for cycle 2 was calculated as 94.57%. In the same way the material was tested for cycle 3 and cycle 4 and still no significant change in photocatalytic efficiency was observed. Figure 10 shows the results obtained by reusability testing up to 4 cycles. It can be seen from the results that the material is highly stable towards treatment of AN dye up to more than 4 cycles of use.

### 3.6. Comparison with the Literature

Data from previous studies using synthesized CuO material were compared with the present study in respect of different parameters such as time, concentration, catalyst dose, light source used and efficiency of degradation. Table 5 shows that approaches described in the present study were more efficient and more valuable for the photocatalytic degradation of AN dye.

## 4. Conclusions

The study used green synthesis of CuO NRs stabilized with Alg-Agar in a biopolymer matrix. The crystalline, structural, morophological and optical properties of the synthesized material were analyzed by using different techniques such as XRD, FTIR, SEM-EDX, TEM, SAED, XPS and UV-VIS. The optical properties of BNC revealed that there is a contraction in band gap value from 2.53 eV in CuO NRs to 2.37 eV in CuO/Alg-Agar BNC. XPS studies revealed the existence of CuO with +2 oxidation and that oxides are formed on the surface of biopolymer matrix. RSM-CCD design was engaged to optimize the maximum response by interaction of two or more variables for statistical results. The degradation of AN was optimised at pH 4, 80 mg L^−1^ AN concentration, 20 mg catalyst dose and irradiation time of 45 min in the presence of visible light irradiation. The photocatalysis reaction shows a pseudo first order kinetic reaction with constant rates of 0.06, 0.09, 0.11, 0.13 min^−1^ with half-life order (t_1/2_) as 9.94, 7.68, 5.87 and 5.33 min for 20, 40,40, 60 and 80 ppm AN concentrations, respectively. The scavenger experiment shows the ^•^OH radical as the major ROS for the photocatalytic degradation of AN. Thus, our CuO/Alg-Agar BNC material will be challenging and interesting material for the degradation of dye in treatment of wastewater.

## Figures and Tables

**Table 1:** Actual actors affecting the photocatalytic degradation of AN by CuO/Alg-Agar BNC and their levels.

Factor	Name	Units	Minimum	Maximum	Coded Low	Coded High	Mean	Std. Dev.
A	Irradiation Time	min	24.89	50.11	−1 ↔ 30.00	+1 ↔ 45.00	37.50	6.36
B	pH		2.32	5.68	−1 ↔ 3.00	+1 ↔ 5.00	4.00	0.8478
C	AN Conc	mg L^−1^	26.36	93.64	−1 ↔ 40.00	+1 ↔ 80.00	60.00	16.96

**Table 2:** FTIR vibrational frequency peaks of CuO/Alg-Agar BNC and corresponding functional groups.

Constituents	Wavenumber (cm^−1^)	Types of Functional Groups	Reference
Agar	3330 2981 1632 1370 1044	-OH stretching -CH stretching C=O stretching C-OH stretching C-O-C stretching	[39]
CuO	3386 1092 870 600	-OH stretching -OH bending -H_2_O rocking Cu-O stretching	[40]
CuO/Alg-Agar BNC	3378 1092 870 600		

**Table 3:** ANOVA for quadratic model.

Source	Sum of Squares	df	Mean Square	F-Value	*p*-Value	
**Model**	249.67	9	27.74	6.57	0.0035	significant
A-Irradiation Time	10.78	1	10.78	2.55	0.1413	
B-pH	9.40	1	9.40	2.23	0.1666	
C-AN Conc	158.02	1	158.02	37.40	0.0001	
AB	0.3002	1	0.3002	0.0710	0.7952	
AC	1.63	1	1.63	0.3868	0.5479	
BC	0.3415	1	0.3415	0.0808	0.7820	
A^2^	1.53	1	1.53	0.3611	0.5613	
B^2^	23.23	1	23.23	5.50	0.0410	
C^2^	38.13	1	38.13	9.02	0.0133	
**Residual**	42.25	10	4.23			
Lack of Fit	12.87	5	2.57	0.4382	0.8069	not significant
Pure Error	29.38	5	5.88			
**Cor Total**	291.92	19				

The standard deviation of the model is 2.06, while correlation values of R^2^ and R^2^_adj_ are 0.8553 and 0.7250, respectively, indicating the correlation between experimental and theortical values of the phototcatalyst’s response.

**Table 4:** Kinetic parameters and their values for pseudo first order kinetic model proposed by Langmuir–Hinshelwood for degradation of AN by synthesized material.

S.N.	AN Concentration (mgL^−1^)	Rate Constant (k_1_) (min^−1^)	Half-Life t_1/2_ (min)	R^2^
1.	20	0.06	9.94	0.99
2.	40	0.09	7.68	0.99
3.	60	0.11	5.87	0.99
4.	80	0.13	5.33	0.99

**Table 5:** Comparison of present study with literature.

Material	Light Source	Time (min)	Concentration (mg L^–1^)	Dosage (mg)	Dye	Efficiency (%)	Reference
Carboxymethyl cellulose/chitosan-CuO	Halide lamp	30	500	30	Acid black	88.60	[59]
β-CD-CuO/ZnO	Halide lamp	180	10	50	Methylene Blue	89.15	[60]
NiO-CuO-RGO	Visible light	60	10	50	Brilliant dye	91	[61]
CuO/γ-Al2O3	Visible LED light	150	0.30	5	Brilliant Red B	90.72	[62]
Ce-doped CuO	Visible light	40	10	10	Methylene Blue	87.72	[20]
Biogenically synthesized CuO	Visible light	90	4	40	Xylenol Orange	87	[63]
CuO/Alg-Agar BNC	Visible solar light	45	80	20	Amaranth	95	Present Study

## Data Availability

Data is contained within the article.

## Supplementary Materials

The following supporting information can be downloaded at: https://www.mdpi.com/article/10.3390/polym15030553/s1, Table S1: Design Table with actual vs. predicted values of photocatalytic efficiency; Table S2: Absorbance vs. irradiation time at different AN concentration.

## References

[1] Muhmood T., Xia M., Lei W., Wang F., Mahmood A. (2018). Fe-ZrO_2_ Imbedded Graphene like Carbon Nitride for Acarbose (ACB) Photo-Degradation Intermediate Study. Adv. Powder Technol..

[2] Muhmood T., Uddin A. (2020). Fabrication of Spherical-Graphitic Carbon Nitride via Hydrothermal Method for Enhanced Photo-Degradation Ability towards Antibiotic. Chem. Phys. Lett..

[3] Shahmoradi F., Taghizadeh M., Taghizadeh A. (2021). Environmental Technology & Innovation Clean Synthesis of Rock Candy-like Metal—Organic Framework Biocomposite for Toxic Contaminants Remediation. Environ. Technol. Innov..

[4] Morajkar P.P., Naik A.P., Bugde S.T., Naik B.R. (2019). Photocatalytic and Microbial Degradation of Amaranth Dye.

[5] Zhang M., Lin K.A., Huang C., Tong S. (2019). Enhanced Degradation of Toxic Azo Dye, Amaranth, in Water Using Oxone Catalyzed by MIL-101-NH2 under Visible Light Irradiation. Sep. Purif. Technol..

[6] Rosu M., Coros M., Pogacean F., Magerusan L. (2017). Graphical Abstract SC. Solid State Sci..

[7] Guerrero-Coronilla I., Morales-Barrera L., Cristiani-Urbina E. (2015). Kinetic, Isotherm and Thermodynamic Studies of Amaranth Dye Biosorption from Aqueous Solution onto Water Hyacinth Leaves. J. Environ. Manag..

[8] Omrani E., Ahmadpour A., Heravi M., Rohani T. (2022). Novel ZnTi LDH/h-BN Nanocomposites for Removal of Two Different Organic Contaminants: Simultaneous Visible Light Photodegradation of Amaranth and Diazepam Journal of Water Process Engineering Novel ZnTi LDH/h-BN Nanocomposites for Removal of Two Different Organic Contaminants: Simultaneous Visible Light Photodegradation of Amaranth and Diazepam. J. Water Process Eng..

[9] Anjaneya O., Shrishailnath S.S., Guruprasad K., Nayak A.S., Mashetty S.B., Karegoudar T.B. (2013). International Biodeterioration & Biodegradation Decolourization of Amaranth Dye by Bacterial Bio Fi Lm in Batch and Continuous Packed Bed Bioreactor. Int. Biodeterior. Biodegrad..

[10] Haritha E., Mohana S., Madhavi G., Elango G., Arunachalam P. (2016). Catunaregum Spinosa Capped Ag NPs and Its Photocatalytic Application against Amaranth Toxic Azo Dye Catunaregum Spinosa Capped Ag NPs and Its Photocatalytic Application against Amaranth Toxic Azo Dye. J. Mol. Liq..

[11] López J., Ortíz A.A., Dominguez D., León J.N.D., De Galindo J.T.E., Hogan T., Gómez S., Tiznado H., Herrera G.S. (2020). Magnetic nanostructured based on cobalt–Zinc Ferrites designed for photocatalytic dye degradation. J. Phys. Chem. Solids.

[12] Hitam C.N.C., Jalil A.A. (2020). A Review on Exploration of Fe_2_O_3_ Photocatalyst towards Degradation of Dyes and Organic Contaminants. J. Environ. Manag..

[13] Sadeghfar F., Zalipour Z., Taghizadeh M., Taghizadeh A., Ghaedi M. (2021). Photodegradation Processes. Interface Sci. Technol..

[14] Li Z., Zhang Z., Wang L., Meng X. (2020). Bismuth Chromate (Bi_2_CrO_6_): A Promising Semiconductor in Photocatalysis. J. Catal..

[15] Byrne C., Subramanian G., Pillai S.C. (2018). Journal of Environmental Chemical Engineering Recent Advances in Photocatalysis for Environmental Applications. J. Environ. Chem. Eng..

[16] Sikiru A., Popoola L.T., Aderibigbe E.I. (2020). Solar Photocatalytic Degradation of Organic Pollutants in Textile Industry Wastewater by ZnO/Pumice Composite Photocatalyst. J. Environ. Chem. Eng..

[17] Karimifard S., Alavi Moghaddam M.R. (2018). Application of Response Surface Methodology in Physicochemical Removal of Dyes from Wastewater: A Critical Review. Sci. Total Environ..

[18] Banerjee S., Pillai S.C., Falaras P., O’shea K.E., Byrne J.A., Dionysiou D.D. (2014). New Insights into the Mechanism of Visible Light Photocatalysis. J. Phys. Chem. Lett..

[19] Alam U., Khan A., Ali D., Bahnemann D., Muneer M. (2018). Comparative Photocatalytic Activity of Sol-Gel Derived Rare Earth Metal (La, Nd, Sm and Dy)-Doped ZnO Photocatalysts for Degradation of Dyes. RSC Adv..

[20] Singh S.J., Chinnamuthu P. (2021). Colloids and Surfaces A: Physicochemical and Engineering Aspects Highly Efficient Natural-Sunlight-Driven Photodegradation of Organic Dyes with Combustion Derived Ce-Doped CuO Nanoparticles. Colloids Surfaces A Physicochem. Eng. Asp..

[21] Uma H.B., Ananda S., Kumar M.S.V. (2021). Electrochemical Synthesis and Characterization of CuO/ZnO/SnO Nano Photocatalyst: Evaluation of Its Application towards Photocatalysis, Photo-Voltaic and Antibacterial Properties. Chem. Data Collect..

[22] Singh J., Soni R.K., Zno-Cuo A. (2021). Colloids and Surfaces A: Physicochemical and Engineering Aspects Efficient Charge Separation in Ag Nanoparticles Functionalized ZnO Nanoflakes/CuO Nanoflowers Hybrids for Improved Photocatalytic and SERS Activity. Colloids Surf. A Physicochem. Eng. Asp..

[23] Rana A., Hasan I., Koo B.H., Khan R.A. (2022). Green Synthesized CeO_2_ Nanowires Immobilized with Alginate-Ascorbic Acid Biopolymer for Advance Oxidative Degradation of Crystal Violet. Colloids Surf. A Physicochem. Eng. Asp..

[24] Chen X., Wu Z., Liu D., Gao Z. (2017). Preparation of ZnO Photocatalyst for the Efficient and Rapid Photocatalytic Degradation of Azo Dyes. Nanoscale Res. Lett..

[25] Rakibul M., Shahriyar S., Kabir A., Farhad F.U. (2021). Colloids and Surfaces A: Physicochemical and Engineering Aspects Synthesis, Characterization and Visible Light-Responsive Photocatalysis Properties of Ce Doped CuO Nanoparticles: A Combined Experimental and DFT + U Study. Colloids Surf. A Physicochem. Eng. Asp..

[26] Cai Y., Yang F., Wu L., Shu Y., Qu G., Fakhri A., Gupta V.K. (2020). Hydrothermal-ultrasonic synthesis of CuO nanorods and CuWO_4_ nanoparticles for catalytic reduction, photocatalysis activity, and antibacterial properties. Mater. Chem. Phys..

[27] Lv Y., Liu J., Zhang Z., Zhang W., Wang A., Tian F. (2021). Green Synthesis of CuO Nanoparticles-Loaded ZnO Nanowires Arrays with Enhanced Photocatalytic Activity. Mater. Chem. Phys..

[28] Sharma S., Kumar K., Thakur N., Chauhan S. (2021). Eco-Friendly Ocimum Tenuiflorum Green Route Synthesis of CuO Nanoparticles: Characterizations on Photocatalytic and Antibacterial Activities. J. Environ. Chem. Eng..

[29] Pavithra N.S., Manukumar K.N., Viswanatha R., Nagaraju G. (2021). Combustion-Derived CuO Nanoparticles: Application Studies on Lithium-Ion Battery and Photocatalytic Activities. Inorg. Chem. Commun..

[30] George A., Antoni D.M., Venci X., Raj A.D., Irudayaraj A.A., Josephine L., Sundaram S.J., Al-mohaimeed A.M., Al D.A., Chen T. (2022). Photocatalytic Effect of CuO Nanoparticles Flower-like 3D Nanostructures under Visible Light Irradiation with the Degradation of Methylene Blue (MB) Dye for Environmental Application. Environ. Res..

[31] Ngoc H., Pansambal S., Ghotekar S., Oza R. (2022). New Frontiers in the Plant Extract Mediated Biosynthesis of Copper Oxide (CuO) Nanoparticles and Their Potential Applications: A Review. Environ. Res..

[32] Francavilla M., Pineda A., Romero A.A., Colmenares J.C., Vargas C., Monteleone M., Luque R. (2014). Efficient and Simple Reactive Milling Preparation of Photocatalytically Active Porous ZnO Nanostructures Using Biomass Derived Polysaccharides. Green Chem..

[33] Roy S., Rhim J.W. (2021). Preparation of Pectin/Agar-Based Functional Films Integrated with Zinc Sulfide Nano Petals for Active Packaging Applications. Colloids Surf. B Biointerfaces.

[34] Thomas M., Naikoo G.A., Sheikh M.U.D., Bano M., Khan F. (2016). Effective Photocatalytic Degradation of Congo Red Dye Using Alginate/Carboxymethyl Cellulose/TiO_2_ Nanocomposite Hydrogel under Direct Sunlight Irradiation. J. Photochem. Photobiol. A Chem..

[35] Tahir C., Zhong Z., Zhou H., Xiao Y. (2020). Constructing Porous Beads with Modified Polysulfone-Alginate and TiO_2_ as a Robust and Recyclable Photocatalyst for Wastewater Treatment. J. Water Process Eng..

[36] Singh R., Bhateria R. (2020). Optimization and Experimental Design of the Pb^2+^ Adsorption Process on a Nano-Fe_3_O_4_—Based Adsorbent Using the Response Surface Methodology. ACS Omega.

[37] Allouss D., Essamlali Y., Amadine O., Chakir A., Zahouily M. (2019). Response surface methodology for optimization of methylene blue adsorption onto carboxymethyl cellulose-based hydrogel beads: Adsorption kinetics, isotherm, thermodynamics and reusability studies. RSC Adv..

[38] Hasan I., Binsharfan I.I., Khan R.A. (2020). L-Ascorbic Acid-g-Polyaniline Mesoporous Silica Nanocomposite for Efficient Removal of Crystal Violet: A Batch and Fixed Bed Breakthrough Studies. Nanomaterials.

[39] Bassi A., Hasan I., Qanungo K., Koo B.H., Khan R.A. (2022). Visible Light Assisted Mineralization of Malachite Green Dye by Green Synthesized Xanthan Gum/Agar@ZnO Bionanocomposite. J. Mol. Struct..

[40] Hasan I., Shekhar C., Bin Sharfan I.I., Khan R.A., Alsalme A. (2020). Ecofriendly Green Synthesis of the ZnO-Doped CuO@Alg Bionanocomposite for Efficient Oxidative Degradation of p-Nitrophenol. ACS Omega.

[41] Suresh S., Karthikeyan S., Jayamoorthy K. (2016). FTIR and multivariate analysis to study the effect of bulk and nano copper oxide on peanut plant leaves. J. Sci. Adv. Mater. Devices.

[42] Hasan I., Walia S., Alharbi K.H., Abu M., Alsalme A., Ahmad R. (2020). Multi-Walled Carbon Nanotube Coupled β -Cyclodextrin/PANI Hybrid Photocatalyst for Advance Oxidative Degradation of Crystal Violet. J. Mol. Liq..

[43] Nagaraj E., Karuppannan K., Shanmugam P., Venugopal S. (2019). Exploration of Bio-Synthesized Copper Oxide Nanoparticles Using Pterolobium Hexapetalum Leaf Extract by Photocatalytic Activity and Biological Evaluations. J. Clust. Sci..

[44] Aroussi S., Dahamni M.A., Ghamnia M., Tonneau D., Fauquet C. (2022). Characterization of Some Physical and Photocatalytic Properties of CuO Nanofilms Synthesized by a Gentle Chemical Technique. Condens. Matter.

[45] Buledi J.A., Pato A.H., Kanhar A.H., Solangi A.R., Batool M., Ameen S., Palabiyik I.M. (2021). Heterogeneous Kinetics of CuO Nanoflakes in Simultaneous Decolorization of Eosin Y and Rhodamine B in Aqueous Media. Appl. Nanosci..

[46] Gupta S.S.R., Kantam M.L. (2021). Finely Dispersed CuO on Nitrogen-Doped Carbon Hollow Nanospheres for Selective Oxidation of Sp^3^. New J. Chem..

[47] Gulati U., Rajesh U.C., Rawat D.S. (2018). RGO@ CuO Nanocomposites From A Renewable Copper Mineral Precursor: A Green Approach For Decarboxylative C(Sp^3^)-H Activation Of Proline Amino Acid To Afford Value-Added Synthons. ACS Sustain. Chem. Eng..

[48] Su X., Feng G., Yu L., Li Q., Zhang H., Song W., Hu G. (2019). In-Situ Green Synthesis of CuO on 3D Submicron-Porous/Solid Copper Current Collectors as Excellent Supercapacitor Electrode Material. J. Mater. Sci. Mater. Electron..

[49] Yang H., Liu J., Wang Y., He C., Zhang L., Mu Y., Li W. (2019). Bioelectrochemistry Bioelectrochemical Decolorization of a Reactive Diazo Dye: Kinetics, Optimization with a Response Surface Methodology, and Proposed Degradation Pathway. Bioelectrochemistry.

[50] Hasan I., Shekhar C., Alharbi W., Khanjer M.A. (2020). Polymers A Highly E Ffi Cient Ag Nanoparticle-Immobilized Alginate-g-Polyacrylonitrile Hybrid Photocatalyst for the Degradation of Nitrophenols. Polymers.

[51] Sharma A.K., Kaith B.S., Tanwar V., Bhatia J.K., Sharma N., Bajaj S., Panchal S. (2019). RSM-CCD optimized sodium alginate/gelatin based ZnS-nanocomposite hydrogel for the effective removal of biebrich scarlet and crystal violet dyes. Int. J. Biol. Macromol..

[52] Bhardwaj B., Kumar R., Singh P.K. (2014). An Improved Surface Roughness Prediction Model Using Box-Cox Transformation with RSM in End Milling of EN 353. J. Mech. Sci. Technol..

[53] Jawad A.H., Alkarkhi A.F.M., Mubarak N.S.A. (2015). Photocatalytic Decolorization of Methylene Blue by an Immobilized TiO_2_ Film under Visible Light Irradiation: Optimization Using Response Surface Methodology (RSM). Desalin. Water Treat..

[54] Hasan I., Bassi A., Alharbi K.H., Binsharfan I.I. (2020). Sonophotocatalytic Degradation of Malachite Green by Nanocrystalline Chitosan-Ascorbic Acid@NiFe_2_O_4_ Spinel Ferrite. Coatings.

[55] Kane S.N., Mishra A., Dutta A.K. (2016). Preface: International Conference on Recent Trends in Physics (ICRTP 2016). J. Phys. Conf. Ser..

[56] Mondol B., Sarker A., Shareque A.M., Dey S.C., Islam M.T., Das A.K., Shamsuddin S.M., Molla M.A.I., Sarker M. (2021). Preparation of Activated Carbon/TiO_2_ Nanohybrids for Photodegradation of Reactive Red-35 Dye Using Sunlight. Photochem.

[57] Pelaez M., Falaras P., Likodimos V., O’Shea K., de la Cruz A.A., Dunlop P.S.M., Byrne J.A., Dionysiou D.D. (2016). Use of Selected Scavengers for the Determination of NF-TiO_2_ Reactive Oxygen Species during the Degradation of Microcystin-LR under Visible Light Irradiation. J. Mol. Catal. A Chem..

[58] Wang Y., Zhang P. (2011). Photocatalytic Decomposition of Perfluorooctanoic Acid (PFOA) by TiO_2_ in the Presence of Oxalic Acid. J. Hazard. Mater..

[59] Xu D., Kong Q., Wang X., Lou T. (2022). Preparation of Carboxymethyl Cellulose/Chitosan-CuO Giant Vesicles for the Adsorption and Catalytic Degradation of Dyes. Carbohydr. Polym..

[60] Yadav R., Chundawat T.S., Surolia P.K., Vaya D. (2022). Photocatalytic Degradation of Textile Dyes Using β-CD-CuO/ZnO Nanocomposite. J. Phys. Chem. Solids.

[61] Sree G.S., Botsa S.M., Reddy B.J.M., Ranjitha K.V.B. (2020). Enhanced UV–Visible Triggered Photocatalytic Degradation of Brilliant Green by Reduced Graphene Oxide Based NiO and CuO Ternary Nanocomposite and Their Antimicrobial Activity. Arab. J. Chem..

[62] Zhao P., Zhao Y., Guo Y., Guo R., Tian Y., Zhao W. (2021). Preparation of CuO/ΓAl_2_O_3_ Catalyst for Degradation of Azo Dyes (Reactive Brilliant Red X–3B): An Optimization Study. J. Clean. Prod..

[63] Gudipati T., Zaman M.B., Singh P., Poolla R. (2021). Enhanced Photocatalytic Activity of Biogenically Synthesized CuO Nanostructures against Xylenol Orange and Rhodamine B Dyes. Inorg. Chem. Commun..

