# Supplementary material for: CuO Nanorods Immobilized Agar-Alginate Biopolymer: A Green Functional Material for Photocatalytic Degradation of Amaranth Dye
Source: Polymers (Basel). 2023 Jan 21;15(3):553. doi: 10.3390/polym15030553 (PMC9921830; doi:10.3390/polym15030553)
Supplement: Supplementary file 1 [file polymers-15-00553-s001.zip › polymers-2131946-supplementary.pdf]

Table S1. Design Table with actual vs predicted values of photocatalytic efficiency.

Std	Run	Irradiation Time (min)	pH	AN Conc. ppm	Actual Value	Predicted Value
1	1	30	3	40	86.5702	86.31
2	2	45	3	40	91.219	89.38
3	3	30	5	40	86.5702	85.45
4	4	45	5	40	87.1901	87.74
5	5	30	3	80	95.4545	94.43
6	6	45	3	80	95.0413	95.69
7	7	30	5	80	91.374	92.74
8	8	45	5	80	93.4401	93.23
9	9	24.8866	4	60	88.4986	88.89
10	10	50.1134	4	60	91.5978	91.88
11	11	37.5	2.31821	60	95.0413	96.29
12	12	37.5	5.68179	60	94.0771	93.50
13	13	37.5	4	26.3641	79.621	80.98
14	14	37.5	4	93.6359	93.1152	92.42
15	15	37.5	4	60	95.7989	91.30
16	16	37.5	4	60	91.2534	91.30
17	17	37.5	4	60	88.4298	91.30
18	18	37.5	4	60	90.6336	91.30
19	19	37.5	4	60	91.1846	91.30
20	20	37.5	4	60	90.6336	91.30

Table S2. Absorbance vs irradiation time at different AN concentration.

AN Concentration (mg L^{-1})	Absrobance Values				
	**10 min**	**20 min**	**30 min**	**40 min**	**50 min**
20	0.3632	0.227	0.151333	0.121067	0.106824
40	0.227	0.165091	0.121067	0.100889	0.086476
60	0.1816	0.139692	0.106824	0.0908	0.078957
80	0.151333	0.121067	0.100889	0.086476	0.07264
